# Sanitary Conditions on the Farm Alters Fecal Metabolite Profile in Growing Pigs

**DOI:** 10.3390/metabo12060538

**Published:** 2022-06-11

**Authors:** Soumya K. Kar, Marinus F. W. te Pas, Leo Kruijt, Jacques J. M. Vervoort, Alfons J. M. Jansman, Dirkjan Schokker

**Affiliations:** 1 Animal Nutrition, Wageningen Livestock Research, Wageningen University & Research, 6708 WD Wageningen, The Netherlands; alfons.jansman@wur.nl; 2 Animal Breeding & Genomics, Wageningen Livestock Research, Wageningen University & Research, 6708 WD Wageningen, The Netherlands; marinus.tepas@wur.nl (M.F.W.t.P.); leo.kruijt@wur.nl (L.K.); 3 Department of Agrotechnology and Food Sciences, Biochemistry, Wageningen University & Research, 6708 WE Wageningen, The Netherlands; 4 Epidemiology, Bio-informatics & Animal Models, Wageningen Bioveterinary Research, Wageningen University & Research, 8200 AB Lelystad, The Netherlands; dirkjan.schokker@wur.nl

**Keywords:** feces, health, immune, pig, metabolomics, nuclear magnetic resonance

## Abstract

The aim of this study was to use fecal metabolite profiling to evaluate the effects of contrasting sanitary conditions and the associated subclinical health status of pigs. We analyzed fecal metabolite profiles by nuclear magnetic resonance (^1^H NMR) from pigs aged 14 and 22 weeks. Pigs kept under low and high sanitary conditions differed in fecal metabolites related to the degradation of dietary starch, metabolism of the gut microbiome, and degradation of components of animal (host) origin. The metabolites that differed significantly (FDR < 0.1) were from metabolic processes involved in either maintaining nutrient digestive capacity, including purine metabolism, energy metabolism, bile acid breakdown and recycling, or immune system metabolism. The results show that the fecal metabolite profiles reflect the sanitary conditions under which the pigs are kept. The fecal metabolite profiles closely resembled the profiles of metabolites found in the colon of pigs. Fecal valerate and kynurenic acid could potentially be used as “non-invasive” biomarkers of immune or inflammatory status that could form the basis for monitoring subclinical health status in pigs.

## 1. Introduction

The management conditions imposed on pigs, including sanitary status, affect the productivity, health, and welfare of pigs [1,2]. Low sanitary conditions (LSC) activate the immune system, reduce growth performance and change nutrient and energy requirements compared to pigs raised under high sanitary conditions (HSC) [3,4]. In a previous publication, we showed how sanitary conditions altered the composition of the microbiome in the colon, and how this affected the metabolome in the colon and the metabolome in the blood of pigs of 13 weeks of age [3]. Based on the results from the previous study by te Pas and colleagues, it was concluded that the microbiome composition and the metabolomes were influenced by the sanitary status and related suboptimal health status of the pigs [3]. Primarily, the metabolic data of the study [3] are useful to investigate the relationships between health, metabolic status, and parameters related to growth performance. Such metabolite data could help to identify biomarkers for (gut) health and performance, as also indicated by Beaumont and colleagues [5]. Fecal biomarkers could help to monitor the effects of the sanitary and associated health status of pigs and support the implementation of interventions to prevent negative effects on productive performance. Furthermore, another study has shown that fecal metabolites could serve as proxy for colon [6]. Therefore, in the present study, fecal metabolite profiles were measured in pigs reared in different sanitary conditions, including pigs being subjected to different vaccination regimes.

## 2. Results

The Feed Conversion Ratio (FCR) of the HSC group was 2.24 and for the LSC group 2.21, with a standard error of the mean of 0.029, and no significant (*p* > 0.05) effect was observed. We obtained global NMR profiles based on fecal samples from pigs in the HSC and LSC groups. The HSC/LSC ratio of metabolite abundances that were significantly different between the two treatments at week 14 and week 22, with bin numbers and metabolite names as well as the expected biological origin of the metabolites, are shown in Table 1.

The number of metabolites that differed significantly between HSC and LSC pigs was higher at week 22 compared to week 14. We observed dietary metabolites such as xanthine, a purine base found in the purine metabolic pathway, whose concentration in the feces of HSC pigs was higher than that of LSC pigs at week 22. Furthermore, in Table 1, metabolites were assigned to microbial metabolism when they should not be derived from diet or from (endogenous) metabolism in any tissue of the pig. With the exception of a propionate compound and uridine derivatives, metabolites derived from the intestinal microbiome, such as UDP-glucose (FDR < 0.05) and valerate-like (FDR < 0.1) compounds, showed significant differences in abundance only between HSC and LSC pigs at 22 weeks of age. Higher concentrations of uridine derivatives were observed in fecal samples of 14-week-old pigs kept in HSC pigs than in LSC pigs. Fecal samples at 22 weeks showed a different ratio in metabolites that are thought to originate from the endogenous metabolism of pigs, i.e., bile acids and kynurenic acid. The concentration of bile acids was higher in the feces of HSC pigs than in the feces of LSC pigs. However, kynurenic acid was higher in the feces of LSC pigs than in the feces of HSC pigs.

## 3. Discussion

The results of the present study show that pigs kept at LSC and HSC differ in their fecal metabolite profiles. Part of the experimental sanitary conditions relates to different vaccination strategies, so the influence of vaccination cannot be completely excluded. Since the results contain data that cannot be related to the effects of vaccination, we conclude that the sanitary conditions applied were the main cause of the observed results. Moreover, the approach chosen for fecal metabolite profiling, i.e., the ^1^H NMR method, is biased, as has been shown for other metabolite profiling techniques, such as liquid and gas chromatography [7]. The results of this study suggest that fecal metabolite profiles are related to the degradation of dietary starch, the metabolism of the gut microbiome, and the degradation of components of animal (host) origin. In our previous publication, redundancy analysis showed that the colon microbiota composition of the gut microbiota of pigs kept under the two sanitary statuses differed greatly [3]. Four bacterial groups in the colon were more abundant in HSC housing than in LSC. These were associated with better gut health, energy supply, SCFA synthesis, and higher productivity. Similarly to our previous publication, we showed that metabolites with different abundance in HSC and LSC fecal samples could be related to either diet, host metabolism, or microbiome composition. In addition, the fecal metabolite profiles strongly resembled the profiles of metabolites found in the colon of pigs [3,5] demonstrating a high similarity between colon and fecal samples, making sampling easier and less burdensome for the animal. The observed metabolites that differed significantly when comparing LSC and HSC were from metabolic processes involved in either maintaining nutrient digestive capacity, including purine metabolism, energy metabolism, bile acid breakdown and recycling, or immune system metabolism.

Purine bases are normally present in the feces of growing pigs [8]. Our results suggest that the intestinal tissues of pigs absorb purine bases to different extents under different sanitary conditions. We observed a higher concentration of xanthine in the feces, which is consistent with our previous work [3], in which we found that the concentration of inosine, a xanthine precursor, was higher in the HSC digesta than in the LSC pig intestinal digesta. During purine metabolism, inosine and xanthine are the upstream metabolites that lead to the synthesis of uric acid. Purine metabolism can occur in different intestinal segments and therefore it is plausible that we observe a spatio-temporal effect of these metabolites, i.e., we observed precursors of purine metabolism more proximally and end products more distally. Based on the higher levels of xanthine in HSC pigs, we hypothesize that HSC pigs are comparatively more efficient than pigs kept at LSC in excreting “undesirable” metabolites known to have negative effects on human physiological status [9]. However, more research is needed to further clarify this postulate in pigs.

We observed significant differences in the abundance of metabolites related to the resident microbiome. The observed differences in HSC and LSC pigs are likely related to differences in the composition of the resident microbiome, as we observed striking differences in the composition and diversity of the colon microbiome in our previous study [3]. Several of the metabolites derived from the microbiome belong to the short-chain fatty acids (SCFAs), such as butyrate and propionate. These metabolites are important as a source of energy for colonocytes [10]. All of these SCFAs were higher in feces from LSC compared with HSC pigs. This could mean that the colonocytes of healthy HSC pigs absorbed more SCFAs than the colonocytes of LSC pigs. Another explanation for this observation is that the microbiome of LSC pigs synthesized more SCFAs than HSC pigs. However, we hypothesize that the functional activities of the microbiome are less efficient in LSC pigs compared to HSC pigs [3], making the alternative explanation less likely. Other metabolites derived from the microbiome, such as glucose derivatives, are associated with cellular energy metabolism. Uridine diphosphate (UDP)-glucose was higher in feces from HSC compared to LSC pigs. This metabolite is known to be involved in carbohydrate metabolism [11], and this metabolite was not significantly different in the colon of pigs in a previous study [3]. It is likely that the fermentation of carbohydrates continues in the hindgut, i.e., rectum, leading to differences in metabolite signatures between colonic digesta and feces. We were able to capture another metabolite from the microbiome that differs between HSC and LSC pigs, namely valerate, a salt or ester of valeric acid. Valerate is a branched SCFA produced from the branched-chain amino acid valine and was higher in HSC pigs than in LSC. Like other branched SCFAs, valerate has been shown to modulate innate immune responses via specific receptors on macrophages and T cells [12,13]. In this and the previous study [3], we observed a higher content of urocanic acid (i.e., Bin number 7.86; uridine derivatives), which is known for its anti-inflammatory activity [14]. Such immune-related metabolites, such as valerate and urocanic acid, are produced in the gut as a result of a local immune response that regulates host gut health. Our observations indicate that HSC pigs elicit a strong local immune response compared to LSC pigs, indicating a stable and alert intestinal immune status. The higher concentrations of these immune-related metabolites in feces may be due to the fact that HSC pigs are not exposed to pathogenic challenges and therefore these are not used and excreted by the host.

Metabolites in feces could be derived from endogenous metabolism in pigs. We observed two host-endogenous metabolites, bile acids and kynurenic acid. Primary bile acids are synthesized by the liver and secreted into the intestinal lumen to form ‘micelles’ and allow the uptake of fatty acids in the intestine [15]. Once the primary bile acids are excreted in the intestine, they are converted to secondary bile acids by colonic bacteria [16]. The size of the bile acid pool is small, which means that bile acids are recycled several times a day [17]. Approximately 95% of bile acids are reabsorbed by active transport in the ileum and returned to the liver, where they are again excreted into the biliary system and gallbladder. Therefore, although not proven, it can be assumed that the bile acids measured in feces are secondary bile acids. Higher levels of bile acids are measured in HSC pigs than in LSC pigs, suggesting that HSC pigs produce a larger pool of bile acids, in turn suggesting a more effective nutrient absorption capacity. Another host-derived metabolite we detected is kynurenic acid, a metabolic product of L-tryptophan, which was detected at higher levels in LSC pigs compared to HSC pigs.

Kynurenic acid has neuroactive and anticytotoxic effects. High urinary levels of kynurenic acid have been found in certain metabolic disorders in humans, such as marked pyridoxine deficiency, which is clinically associated with a beneficial effect on a number of gastrointestinal diseases, especially ulcers, colon obstruction, or colitis [18]. In humans, the ratio of kynurenic acid to tryptophan is used as an indicator of inflammatory status [19]. It is uncertain whether the higher fecal concentration of kynurenic acid in LSC pigs could be related to a difference in the local immune response of HSC and LSC pigs in the present study. It is noteworthy that in humans and animal models, higher fecal kynurenic acid concentrations are associated with pathophysiological conditions such as systemic lupus erythematosus [20] and inflammatory bowel disease [21,22,23]. Further studies are warranted to clarify the presence of kynurenic acid in feces and highlight its importance in the health status of pigs.

## 4. Materials and Methods

The experimental set-up to generate low sanitary conditions (LSC) and high sanitary conditions (HSC) has previously been described [3,4]. Briefly, we created a contrast in sanitary status of weaned piglets from four weeks of age to slaughter weight. The LSC pigs were housed in a low sanitary environment created by frequent application of fresh manure from other pigs at the Swine Innovation Centre of Wageningen University & Research. The fresh manure, a mix of fresh manure of younger and older pigs and weaned piglets, was spread in the LSC pens to enhance the contrast in sanitary status between treatments, and the LSC pigs did not receive vaccinations against a number of relevant pathogens for pigs. In contrast, HSC pigs were housed in a more hygienic environment and were vaccinated against a range of relevant pathogens. Per pen (ten pigs), 1 feeder was used, and feed and water were offered ad libitum. Feed was given in the form of pellets via a computer-controlled automated system (Fancom Multiphase; Fancom B.V., Panningen, The Netherlands) that recorded the amount of feed given per pen per day. At the end of each phase (starter, grower, and finisher), body weight and feed residue were collected and pigs were weighed per pen to determine feed intake per pen in each phase and subsequently calculate FCR. The computerized feeding system was calibrated before the start of the experiment and after each phase. We collected fecal samples from six animals (one pig out of ten pigs per pen) per experimental treatment at 14 and 22 weeks of age (starter and finisher phase, respectively), and measured metabolites in these samples using ^1^H NMR metabolomics. In general, fecal metabolite profiles represent: metabolites from the diet; intermediates of dietary nutrients formed during nutrient hydrolysis and nutrient fermentation in the digestive tract; metabolites related to intestinal microbiome metabolism; and metabolites from systemic metabolism of the host [24,25,26].

We established initial global NMR profiles based on fecal samples from pigs in the HSC and LSC groups. The data were first preprocessed by a so-called binning procedure, to reduce the dimensionality of the data. We set the bin size, i.e., the value interval, to 0.01 of the NMR data, resulting in a total of 1000 bins per sample. Thus, broader peaks were divided into multiple bins. Twenty bins were removed because they interfered with the measurements. A second filtering step was performed based on the 980 bins, with the threshold set at 100,000. This resulted in 768 bins that were used for further analysis. This further downstream analysis encompassed a linear model, with the fixed effects of age, i.e., week 14 or 22, and treatment, i.e., LSC or HSC. The *p* values that were generated by this analysis were subsequently adjusted for multiple testing correction by employing a false discovery rate (FDR). A threshold FDR value of <0.1 was used, meaning that 10% of the statistically significant values are possible false positives. Where possible, the bins were annotated with metabolite names, and the metabolites with different ratio in LSC compared to HSC were biologically annotated. Details of the annotation method and biological interpretation can be found in te Pas et al. [3].

## 5. Conclusions

Overall, fecal metabolite profiles provide a better understanding of the subclinical health status and physiology of pigs kept under different sanitary conditions. These fecal metabolite profiles show strong similarities to the metabolite profiles in the colon digesta of pigs, which is consistent with previous studies [3,6]. In addition, fecal valerate content could potentially be used as a biomarker for porcine immune status, and kynurenic acid could be used as a biomarker for porcine inflammatory status, although further evidence is warranted. The use of biomarkers could provide the basis for either monitoring subclinical health status in pigs or formulating targeted nutritional interventions aimed at (re)balancing the immune system in pigs.

## Figures and Tables

**Table 1:** Metabolites showing different ratios in feces of pigs kept in high (HSC) or low sanitary conditions (LSC) at 14 and 22 weeks of age.

Expected Origin	Bins	Metabolite	Week 14		Week 22	
			Ratio HSC/LSC	FDR ^1^	Ratio HSC/LSC	FDR
Diet	7.93	Xanthine	1.53	ns ^2^	1.41	<0.1
Microbiome	1.52	Butyrate	0.76	ns	0.78	<0.1
Microbiome	0.88	Butyrate	0.65	ns	0.75	<0.1
Microbiome	2.18	Butyrate and propionate	0.81	ns	0.86	<0.1
Microbiome		Mean butyrate ^3^	0.74	ns	0.79	<0.1
Microbiome	1.06	Propionate	0.73	<0.05	0.73	<0.05
Microbiome		Mean propionate ^3^	0.77	ns	0.79	<0.1
Microbiome	5.64, 5.63, 5.62	UDP-glucose ^4^	1.15	ns	4.76	<0.05 ^5^
Microbiome	0.84	Valerate (tentative annotation) ^6^	0.84	ns	1.37	<0.1
Microbiome	5.94, 6.13, 6.12, 6.11, 7.89, 7.86	Uridine derivates	2.27	<0.1 ^5^	1.36	ns
Endogenous (host)	0.77, 0.76, 0.75, 0.74, 0.73	Bile acids (tentative)	0.64	ns	1.54	<0.1 ^5^
Endogenous (host)	6.65 (7.82, 7.68, 7.49)	Kynurenic acid	0.80	ns	0.62	<0.1 ^5^

^1^ False discovery rate; ^2^ not significant; _3_ mean value of the bins related to the metabolite; ^4^ UDP-glucose: uridine diphosphate-glucose; ^5^ mean FDR over bins; ^6^ tentative annotation: possible closest annotation of the metabolite.

## Data Availability

The NMR data that support the findings of this study are openly available at Zenodo; https://doi.org/10.5281/zenodo.5783902 (3 May 2022).
